# Differentiating the incidence and burden of HIV by age among women who sell sex: a systematic review and meta‐analysis

**DOI:** 10.1002/jia2.26028

**Published:** 2022-10-27

**Authors:** Marie C. D. Stoner, Katherine B. Rucinski, Carrie Lyons, Sue Napierala

**Affiliations:** ^1^ Women's Global Health Imperative RTI International Berkeley California USA; ^2^ Social and Behavioral Interventions Program, Department of International Health Johns Hopkins Bloomberg School of Public Health Baltimore Maryland USA; ^3^ Department of Epidemiology Johns Hopkins Bloomberg School of Public Health Baltimore Maryland USA

**Keywords:** adolescent girls and young women, HIV incidence, HIV prevalence, meta‐analysis, systematic review, young women who sell sex

## Abstract

**Introduction:**

Young women who sell sex (YWSS) are at heightened risk of HIV acquisition and transmission and are among the least engaged in HIV services. There is insufficient epidemiologic evidence characterizing the burden of HIV among YWSS, particularly as compared to older WSS. These data are needed to design and tailor effective HIV prevention and treatment programmes for this population.

**Methods:**

We conducted two parallel systematic reviews and meta‐analyses to define both the immediate and long‐term HIV risks for YWSS, including among women engaged in sex work, survival sex and transactional sex. In the first review, we identified and synthesized published studies of HIV incidence comparing estimates for cisgender women ≤24 years of age versus >24. In the second review, we identified and synthesized studies of HIV prevalence, comparing estimates for cisgender women who initiated selling sex <18 versus ≥18 years. In both reviews, we completed a search of four databases for articles in any language and any geographic area published from 1 January 1980 until 12 February 2021. Included articles were assessed for quality and a random effects model was used to calculate pooled effect estimates for each review.

**Results and Discussion:**

We identified 12 studies for the HIV incidence review and 18 studies for the HIV prevalence review. In a meta‐analysis, HIV incidence was elevated in younger (5.3 per 100 person‐years [PY]; 95% confidence interval [CI]: 3.5, 7.1) compared to older women (2.8 per 100 PY; 95% CI: 1.7, 3.9), although CIs overlapped. HIV prevalence among those who initiated selling sex <18 years of age (28.8; 95% CI: 18.9, 38.7) was higher than those who initiated later (20.5; 95% CI: 12.4, 28.6).

**Conclusions:**

These companion reviews offer an important perspective on the relative HIV risk of engaging in selling sex at a younger age. Our findings highlight the unique and intersectional challenges YWSS face, and the importance of ensuring that health services are tailored to meet their specific needs. Research and programming should routinely stratify data into meaningful age bands to differentiate and intervene within this population.

## 1 INTRODUCTION

Due to the increased availability of biomedical technologies for the prevention and treatment of HIV, there have been significant decreases in HIV incidence, morbidity and mortality observed globally [1, 2]. Despite these successes, epidemiologic gains have not been evenly distributed across populations. As a result, the global HIV agenda has seen an increased focus on improving HIV prevention and treatment for key and priority populations who remain at heightened risk of HIV acquisition and transmission and are marginalized from existing services, including both young people and women who sell sex (WSS).

Key populations, including sex workers, men who have sex with men, transgender persons and people who use drugs, have among the highest risk of contracting and transmitting HIV irrespective of the epidemic type or local context and frequently lack adequate access to services [3]. Other vulnerable populations who have also been prioritized by the global HIV response include adolescent girls and young women ages 15–24, who are particularly vulnerable to HIV infection in Southern and Eastern Africa [3]. While young women and WSS are often distinguished as distinct populations in both programmatic and research settings, there are substantial overlapping vulnerabilities. Between 20% and 40% of WSS first initiate sex work during adolescence and a review of HIV incidence in young women in 10 countries found that some of the highest incidence rates were among female sex workers aged 18–24 years in South Africa (13.20 per 100 person‐years [PY]) and in Zimbabwe (10.80 per 100 PY) [4, 5]. Therefore, addressing the intersectional needs of young women who sell sex (YWSS) is critical for optimizing HIV prevention and treatment, meeting UNAIDS 2025 targets to ensure that 95% of women and girls of reproductive age have their HIV and sexual and reproductive healthcare service needs met, and advancing the global agenda of ending HIV/AIDS [6, 7]. Achieving these goals will also require efforts that address the underlying social and structural determinants that drive HIV acquisition and transmission for this population [8, 9].

Evidence quantifying the burden of HIV among YWSS across settings is limited, and few published studies explore the longer‐term epidemiologic implications for women who initiate selling sex at a young age, precluding a full understanding of HIV incidence and prevalence for WSS during adolescence and early adulthood. Moreover, differences in HIV risk may vary by types of sexual exchange, including formal sex work, survival sex or transactional sex, each of which typically reflects different motivations, partner power dynamics and economic circumstances [10, 11, 12]. Finally, young women under the age of 18 cannot be legally defined as sex workers and, therefore, are largely excluded from epidemiologic research due to ethical concerns and legal constraints [4, 13]. These gaps in evidence challenge effective resource allocation and service delivery, and data are urgently needed to better assess and meet the otherwise unmet HIV prevention and treatment needs of YWSS.

The objective of this research was to define both the immediate and long‐term HIV‐related risks for YWSS. To do so, we conducted two systematic reviews in parallel, responding to two related research questions. The first review and meta‐analysis aimed to assess whether YWSS have a higher HIV incidence as compared to older WSS. The second review aimed to determine if young age at initiation of sex work was associated with a higher HIV prevalence as compared to those who initiated sex work at an older age. The findings of the two reviews are presented and synthesized together to provide complementary insights into HIV risks related to young age among WSS.

## 2 METHODS

**Figure 1:**
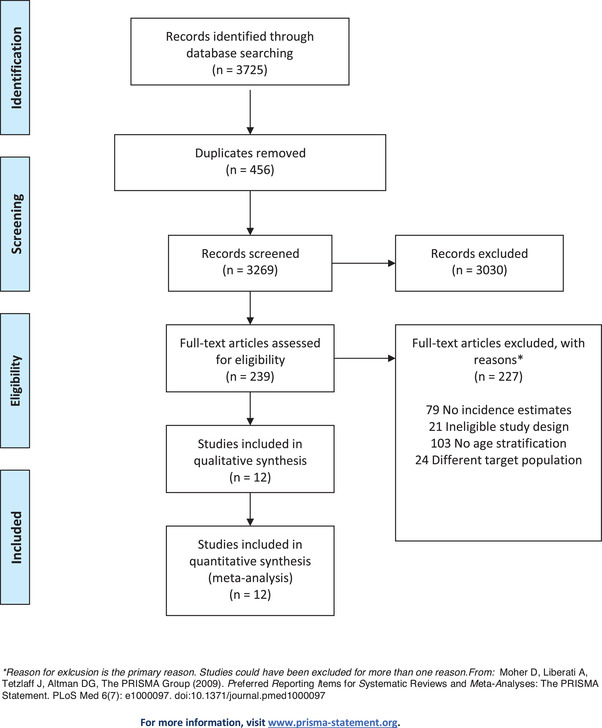
HIV incidence review inclusion criteria.

**Figure 2:**
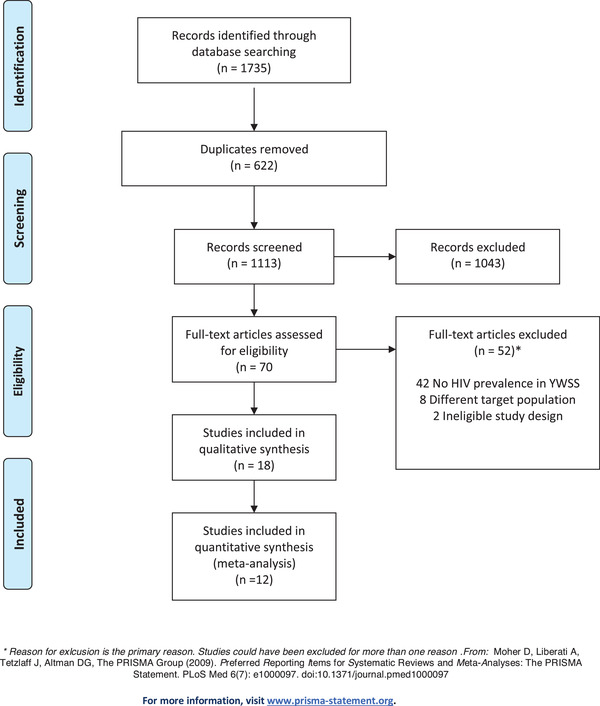
HIV prevalence review inclusion criteria.

We conducted two independent systematic reviews utilizing the standard protocol for Preferred Reporting Items for Systematic Reviews and Meta‐Analysis (PRISMA; see Figures 1 and 2 for the screening flow diagram and Appendix S2 for the PRISMA checklist) [14]. Reviews were conducted concurrently. In the first review, we identified and synthesized all published studies of HIV incidence among WSS that reported disaggregated estimates for women ≤24 years of age (hereafter referred to as the “HIV incidence review”). The categorization of young women under 25 years was selected to align with the UNAIDS definition of adolescent girls and young women which is 15–24 years of age [1]. In the second review, we identified and synthesized all published studies of HIV prevalence among WSS that reported disaggregated estimates for women who initiated selling sex ≤18 years of age (hereafter referred to as the “HIV prevalence review”). We used a similar methodological approach for each review and present our modifications to eligibility criteria and search strategy which differentiate each research question. Meta‐analytic methods were used for both reviews.

### 2.1 Eligibility criteria

Studies published from all countries were eligible for inclusion without restriction by geographic region. Eligible studies were those that (1) included cisgender women who sell sex, including sex work (street‐based, venue‐based, those working at sex work establishments), cisgender women engaged in survival sex work, and cisgender women and girls under the age of 18 engaged in transactional sex; (2) reported sufficient detail on sampling methodology, study design, methods of evaluation and analysis of results; (3) included ≥20 individuals who identified as women or girls and were not limited to men or transgender populations; and (4) were published between 1 January 1980 and 12 February 2021. Systematic reviews and conference abstracts were excluded.

While survival sex, sex work and transactional sex represent different types of commodified sex, they have all been associated with increased risk of HIV and are motivated by different factors which can influence risk [10, 11, 15, 16, 17, 18]. Further, young women ages 18 years and older are considered adults and, therefore, fully able to determine their engagement in the sale of sex; however, the sale of sex by any person under the age of 18 is defined under international law as sexual exploitation [13]. Definitions of YWSS (e.g. sex work, women engaged in survival sex work and transactional sex) were based on the definitions used by the authors of the studies and were all included to represent a fairly wide definition of any type of sexual exchange that could include YWSS over time. Transactional sex was defined based on studies that used this term (typically as the exchange of sex for money, drugs or other gifts) and was included because transactional sex has been associated with a higher risk of HIV among young women and is often used as a proxy for selling sex for those legally under age 18 [11, 13, 19]. Survival sex was defined based on the use of this term in the study of interest.

### 2.2 Methodology unique to HIV incidence review

In addition to meeting the aforementioned eligibility criteria, studies were required to report biologically measured estimates of incident HIV infection. These included both prospective and retrospective longitudinal cohort studies as well as cross‐sectional studies where HIV incidence was captured using an incidence assay (e.g. BED capture enzyme immunoassay). Randomized trials reporting the effects of intervention were included if data were disaggregated by the study arm; in this case, we reported incidence data from the control arm only. Additionally, studies were required to include young women ≤24 years of age and present results stratified by age. We extracted available quantitative data related to HIV incidence for each study. Key variables were extracted overall and by age strata, and included sample size, person time contributed, number of HIV seroconversions and HIV incidence per 100 PY. For studies where person time was not reported, we extracted risk ratios as reported in the study.

### 2.3 Methodology unique to HIV prevalence review

Studies eligible for inclusion were those that met the eligibility criteria above and reported HIV prevalence, as measured by self‐report or through biological testing. All quantitative study designs were eligible. For randomized trials testing interventions, we extracted pre‐intervention measures of HIV prevalence. Studies were also required to report the age at which selling sex was initiated, include women who initiated selling sex at ≤18 years of age and to present data stratified by age at initiation of selling sex. We extracted all available quantitative data related to HIV prevalence, including sample size, the number living with HIV, HIV prevalence and HIV prevalence odds ratio.

### 2.4 Study selection

A search of Pubmed, EMBASE, Web of Science and Sociological Abstracts databases was conducted for articles in any language and in any geographic area published from 1 January 1980 until 12 February 2021. Abstracts not in English were translated via Google Translate [20]. These databases were selected *a priori* as those most likely to contain relevant citations. The search strategies were piloted to assess whether pre‐identified relevant studies had been captured and were refined accordingly. The final search strategies are described in detail in Appendix S3.

Abstracts were imported from each database, duplicates removed and then screened for eligibility based on title and abstract by two independent reviewers using Covidence online software [21]. Articles flagged for inclusion by one or both reviewers underwent a full‐text review. The final decision for inclusion was based on an agreement between the two reviewers, with a third reviewer available in the event of a discrepant decision by the first two reviewers. We also conducted a hand search of references of the citations which met inclusion criteria, to identify any additional relevant studies. We did not identify any additional articles this way, but this provided confirmation that we had included all relevant articles. Descriptive data about the study and sample were extracted, including study design, methods, type of analysis, population/inclusion criteria, the definition of selling sex, age groups reported, time period, geographic location and any other relevant outcomes. We also extracted information relevant to either HIV incidence or HIV prevalence, depending on the outcome.

### 2.5 Quality assessment and risk of bias

We assessed the strength of each included study using a modified quality assessment tool, the Global HIV Quality Assessment Tool for Data Generated through Non‐Probability Sampling (GHQAT) [22]. The tool was specifically developed to evaluate the quality of HIV epidemiologic evidence generated using non‐probability methods, which is common in research studies with key populations. Members of the Global HIV Research Group that supported the development of the tool include collaborators from the Johns Hopkins School of Public Health, The United States Agency for International Development (USAID), The Global Fund to Fight AIDS, Tuberculosis, and Malaria, and UNAIDS. Based on the National Heart, Lung, and Blood Institute's Quality Assessment Tool for Observational Cohort and Cross‐Sectional Studies, the tool evaluates study design, implementation and the strength of core epidemiologic indicators common in HIV‐related key populations research. Each study was scored using the GHQAT by two independent reviewers. Studies received a total score which comprised an evaluation of study design, study implementation, and indicator‐specific criteria. For the HIV incidence review, measures of reported HIV incidence were also graded; for the HIV prevalence review, measures of HIV prevalence were graded. Overall scores were then assessed as good, fair, or poor based on the tool's established criteria. Studies with an overall score of “poor” were excluded from the meta‐analyses in sensitivity analysis to assess the undue influence of these observations on results and findings.

### 2.6 Data synthesis and statistical analysis

The primary measure for the incidence review was HIV incidence per 100 PY. The primary measure for the prevalence review was HIV prevalence. Data were organized both in tabular form and graphically using forest plots. Where 95% confidence intervals (CI) were not reported, asymptotic 95% CIs were constructed for each point estimate using Rothman's EpiSheet [23]. Age‐stratified estimates for each review were visually inspected to assess potential trends by age. Sub‐analyses were conducted to examine how estimates varied across geographical region and definition of YWSS (e.g. sex work, women engaged in survival sex work and transactional sex).

For the incidence review, we conducted a meta‐analysis to calculate the overall HIV incidence rate for both younger and older age groups separately. Given the heterogeneity in age groups that were reported across studies, we categorized incidence rates as occurring among YWSS less than 25 years and among those greater than or equal to 25 years. Incidence rates for multiple age groups above age 25 were combined within studies (e.g. if both 30–39 years and 40–49 years were reported in a single study), and 95% CIs were recalculated for these combined groups. Five estimates were reported for YWSS ages 20–29 years. Given that these estimates did not explicitly fit within either age category (<25 vs. ≥25), they were reported as a separate group. Two additional studies reported an estimate for ages 12–25 years and were not included in the meta‐analysis because it overlapped with the cut‐off (≥25). We conducted a sensitivity analysis including these two studies. We found that when recalculating the incidence estimate, the results were similar to when these studies were not included (∼0.50 increase in estimate). We used a random effects model to calculate a pooled incidence rate. A weighted average of the pooled estimates was calculated using a random intercept model to account for between‐study variation [24, 25].

For the prevalence review, we similarly conducted a meta‐analysis, dichotomizing all point estimates of HIV prevalence as those that were captured among women who initiated selling sex at less than 18 versus 18 years or older. This cut point was chosen based on the legal age of consent and to maximize fidelity to included studies, most of which similarly dichotomized prevalence estimates at 18 years. We used a random effects model to calculate a pooled prevalence estimate, using the same approach as the incidence review [24, 25]. Age was routinely reported as a categorical variable across the studies that were included in these analyses, with HIV incidence and prevalence reported for each age category. Therefore, we report age categories and not continuous measures of age throughout.

### 2.7 Role of the funding source

This study was funded by the National Institute of Mental Health. The study sponsor had no role in the study design, implementation or the decision to submit the paper for publication.

## 3 RESULTS AND DISCUSSION

### 3.1 HIV incidence review

After the exclusion of duplicate citations, a total of 3725 studies were identified from four databases. The title and abstracts were screened for eligibility, and 239 were retained for full‐text review. Reasons for exclusion are reported in Figure 1. A total of 12 studies were eligible for inclusion [26, 27, 28, 29, 30, 31, 32, 33, 34, 35, 36]. Only 11 studies were included in the meta‐analysis because one study included age stratification which overlapped with our age cut‐off. This study was not included in our primary analysis but was included in the sensitivity analysis. These studies are summarized in Table 1, grouped by geographical region. Studies were conducted in sub‐Saharan Africa (*N* = 6) and in Asia (*N* = 6). Most studies were prospective (*N* = 9) or retrospective cohort studies (*N* = 1), and two were cross‐sectional studies that used an incidence assay. Of the 12 studies, seven defined WSS as self‐reported commercial sex work, but one of these also measured “indirect sex work” or transactional sex. Three studies used definitions of transactional sex. The last two studies were not explicit in how they defined selling sex or sexual exchange.

**Table 1:** Study characteristics and estimates of HIV incidence by age

Paper ID	First author's last name	Location	Study design	How is SW defined	Time period	Age stratification	*N*	Number of seroconverters	Person years	HIV incidence (per 100 PYR)	HIV incidence (per 100 PYR) (%)
1	Vandepitte et al. 2013	Kampala, Uganda	Prospective cohort	Not defined	April 2008–March 2011	≤25 years	291	26	493	0.0527	5.27
						26–34 years	297	15	549	0.0273	2.73
						>35 years	58	1	104	0.096	0.96
						ALL	646	42	1147	0.0366	3.66
2	Braunstein et al. 2011	Kigali, Rwanda	Prospective cohort	“High risk of HIV exposure” defined as having exchanged sex for money at least once in the last month and/or currently having sex with multiple partners plus having sex at least twice per week	Oct 2006–Dec 2008	18–20	35	0		Undefined	Undefined
						21–24	143	8	320	0.03	2.50
						25–29	131	5	208.3	0.024	2.40
						30–34	52	6	133.3	0.045	4.50
						ALL (18–46)	361	19	665.5	0.029	2.90
3	Hargreaves et al. 2016	Zimbabwe	Prospective cohort	Women using FSW services	September 2009–March 2014	12–25	223	27	250.0	0.108	10.80
						26–35	262	32	299.1	0.107	10.70
						36+	118	8	133.3	0.06	6.00
						Overall	605	67	686	0.098	9.80
4	Kanki‐Lancet et al. 1994	Dakar, Senegal	Prospective cohort	Self‐identified sex workers	Feb 1985–Feb 1993	20–29	N/A	19	1883.02	0.0101	1.01
						30–39	N/A	15	1859.32	0.0081	0.81
						40–49	N/A	10	373.07	0.0268	2.68
						ALL	1277	46	4141	0.0111	1.11
5	Kilburn et al., 2018	Mpumalanga Province, South Africa	Prospective cohort	Transactional sex, defined as whether a young woman said that she felt that she had to have sex with a male partner as he gave her money or gifts	2011–2016	ALL (13–20)	466	23	N/A	0.05	5.00
				Infrequently received money/gifts (categorical)		ALL (13–20)	234	11	N/A	0.048	4.80
				Frequently received money/gifts (categorical)		ALL (13–20)	232	12	N/A	0.053	5.30
6	Naicker et al. 2015	Kwazulu Natal, South Africa	Prospective cohort		2004/2007	18–24	N/A	8	59.36	0.13	13.48
						> = 25	N/A	10	248.35	0.004	4.03
7	Kilmarx et al. 1998	“Upper northern” Thailand	Prospective cohort	Women had to be registered as commercial sex workers	Oct 1991–December 1994	≤ 19	N/A	14	122.3	0.114	11.4
						20–29	N/A	11	270.8	0.041	4.1
						≥30	N/A	5	301.4	0.017	1.7
						ALL	285	30	694.5	0.043	4.3
8	Morineau et al. 2011	Indonesia	Cross‐sectional analysis. (Used an incidence assay)	Women who sell sex as part or all of their income. Women were recruited from establishments where commercial sex is either solicited or takes place	August 2007–November 2007	<20	13	3	93.8	0.032	3.2
						20–29	153	28	823.5	0.034	3.4
						≥30	63	14	518.5	0.027	2.7
						ALL	229	45	1451.6	0.031	3.1
9	Saphonn et al. 2005	Cambodia	Cross‐sectional analysis. (Used an incidence assay)	Commercial sex workers were defined as brothel‐based commercial sex workers, and IDSWs were defined as women working as beer promotion girls or as bar, karaoke or massage girls		Commercial sex all years					
						14–19	1193	N/A	N/A	0.08	8.16
						20–29	3114	N/A	N/A	0.09	8.96
						30–39	301	N/A	N/A	0.08	7.79
						ALL		N/A	N/A	0.10	9.79
						Indirect sex workers all years					
						14–19	953	N/A	N/A	0.05	6.17
						20–29	2613	N/A	N/A	0.04	6.18
						30–39	226	N/A	N/A	0.01	3.44
						ALL		N/A	N/A	0.04	6.65
10	Couture et al. 2011	Phnom Penh, Cambodia	Prospective cohort	“Engaged in transactional sex (sex in exchange for money, goods, services, or drugs) within the last 3 months”	June 2007–June 2008	16–18	11	0	9	0	0
						19–24	45	2	35	0.057	5.71
						16–24	56	2	46	0.0435	4.3
						25–29	51	1	40	0.025	2.50%
						16–29 (ALL)	123	3	84	0.036	4
11	Gray et al., 1999	Chiang Rai, Thailand	Retrospective cohort study	Sex work was defined as being registered with the Ministry of Public Health as a sex worker (a requirement during the time of the study)	1989–1993	14–19	48	36	N/A	14.9 per 100 person months (pm)	14.9 per 100 pm
						20–29	48	28	N/A	10.5 per 100 pm	10.5per 100 pm
						ALL (14–35)	96	64	377.6 Person months	12.6 per 100 pm	12.6 per 100 pm
12	Su et al., 2016	Kaiyuan, Yunnan Province China	Prospective cohort	Self‐reported engaging in commercial sex in the last 3 months	March 2006–November 2013	≤ 25	594	14	13723	0.001	1.02
						>25	564	19	1733.3	0.010	1.10

Overall HIV incidence ranged from 1.05 per 100 PY in Thailand [28] to 10.8 per 100 PY in Zimbabwe [29]. All studies were published between 1994 and 2018, with seven studies published since 2010. Estimates from studies that used definitions of transactional sex ranged from 2.5 [27] to 6.2 [36] per 100 PY for younger groups, while estimates from studies that used definitions of commercial sex ranged from 1.0 [30] to 11.1 per 100 PY for younger age groups [33]. In studies with an older comparison group (*N* = 11), the incidence was higher in younger compared to older women in six studies, comparable in three studies and higher in older women compared to younger women in two studies (Table 1). Among studies in African countries with an older comparison group (*N* = 5), the incidence was higher in younger compared to older women in two studies, comparable in one study and higher in older women compared to younger in two studies. Among studies in Asian countries (*N* = 6), the incidence was higher in younger compared to older women in four studies and comparable in two studies.

In a meta‐analysis of pooled estimates by age group, the incidence rate for young women below the age of 25 years was 5.3 per 100 PY (95% CI: 3.5, 7.1; Table 3 and Appendix S1 Figure 1). There was substantial heterogeneity between studies (*X* ^2^ = 38.62, degrees of freedom = 8, *p*<0.001) with 79.3% of the variation across studies estimated to be due to heterogeneity rather than chance. In a sensitivity analysis, where we included YWSS ≤25 (two additional studies), the incidence rate for young women less than or equal to 25 years was 5.4 per 100 PY (95% CI: 3.3, 7.4; Appendix S1 Figure 2). Among women greater than or equal to 25 years of age, the incidence rate was 2.8 (95% CI: 1.7, 3.9; Appendix S1 Figure S3) and was similar in a sensitivity analysis excluding two estimates (2.8 per 100 PY; 95% CI: 1.7, 3.9; Appendix S1 Figure S4). Again, there was substantial heterogeneity between studies (*X* ^2^ = 47.02, degrees of freedom = 7, *p*<0.001, *I* ^2^ = 85.1%). We estimated the incidence rate for young women aged 20–29 years to be 4.4 (95% CI: 1.3, 7.4; Appendix S1 Figure S5), again with substantial heterogeneity (*X* ^2^ = 222.91, degrees of freedom = 4, *p*<0.001, *I* ^2^ = 98.2%).

### 3.2 HIV prevalence review

After the exclusion of duplicate citations, a total of 1113 studies were identified from the four databases. The title and abstracts were screened for relevance, and 70 were retained for full‐text review (Figure 2). Reasons for exclusion are reported in Figure 2. A total of 18 studies were eligible for inclusion. These studies are summarized in Table 2, grouped by geographical region [17, 37, 38, 39, 40, 41, 42, 43, 44, 45, 46, 47, 48, 49, 50, 51, 52, 53]. Most studies were in Asia (India *N* = 4, Nepal and Thailand *N* = 2), North America (Canada *N* = 2, the United States = 2 and Mexico), sub‐Saharan Africa (Lesotho and Rwanda) or Latin America (Ecuador and Guatemala), with one study in Ukraine and one study in Iran (Table 2). Almost all studies were cross‐sectional (*N* = 16) but one study was a randomized trial and another was a cohort study. Definitions of WSS varied; nine studies used definitions of formal sex work, one used survival sex and eight studies used definitions of transactional sex.

**Table 2:** Study characteristics and estimates of HIV prevalence by sex work initiation

Paper ID	First author's last name	Location	Study design	How is selling sex defined	Data collection period	Age	Duration of sex work	N	# HIV positive	HIV prevalence (%)	OR	OR CI	AOR	AOR CI
1	Goldenberg et al., 2014	Vancouver, Canada	Cross‐sectional	Exchanged sex for money in the last 30 days	2010–2011	<16	N/A	N/A	N/A	N/A	N/A	N/A	1.88	1.03, 3.42
						<18	N/A	193	38	19.69	N/A	N/A	2.49	1.35, 4.64
						18+	N/A	315	19	6.03	N/A	N/A	1.0 [ref]	
2	Shannon et al., 2007	Vancouver, Canada	Cross‐sectional	This study focused on sex workers engaged in survival sex work, defined as women who exchange sex for money, drugs or shelter as a means of daily survival	2004	<14	N/A	42	13	31.0	1.33	0.78, 2.27	N/A	N/A
						<16	N/A	70	18	25.7	1.1	0.56, 2.19	N/A	N/A
						<18	N/A	93	30	32.3	2.29	1.16, 4.53	1.8	1.3, 2.2
						18+	N/A	105	22	21.0	1.0 [ref]	N/A	1.0 [ref]	N/A
3	Goldenberg et al., 2012	Tijuana, Mexico and Ciudad Juarez, Mexico	Cross‐sectional	Sex work defined as selling or trading sex for money, drugs or other goods. Underage sex work is defined as “selling/trading sex before 18”	October 2008–July 2010	<18	15 years	250	13	5.20	N/A	N/A	N/A	N/A
						18+	13 years	360	22	6.10	N/A	N/A	N/A	N/A
4	Suratt et al., 2012	Miami, Florida, USA	Cross‐sectional	Traded sex for money or drugs at least three times in the past 30 days	May 2007–June 2010	<18	N/A	230	N/A	N/A	N/A	N/A	2.103	1.25, 3.54
						18+	N/A	332	N/A	N/A	N/A	N/A	1.0 [ref]	N/A
5	Footer et al., 2020	Baltimore, Maryland	Cohort	Sold or traded oral, vaginal or anal sex for money or things like food, drugs or favours	2014–2015	<18	N/A	53	6	11.3	N/A	N/A	N/A	N/A
						> = 18	N/A	197	7	3.6	N/A	N/A	N/A	N/A
6	Limpakarnjanarat et al., 1999	Chiang Rai, Thailand	Cross‐sectional	Self‐reported involvement in sex work. No other description of sex work	1991–1994	<15	N/A	69	40	57.97	2.0	1.1, 3.9	N/A	N/A
						15+	N/A	430	120	27.9	1.0 [ref]	N/A	N/A	N/A
7	Silverman et al., 2006	Mumbai, India	Cross‐sectional analysis (medical records review)	Victims of human sex trafficking who were performing sex work against their will at brothels	Dec 2002–July 2005	<15	N/A	23	9	39.10	N/A	N/A	N/A	N/A
						15–16	N/A	43	11	25.60	N/A	N/A	N/A	N/A
						17–18	N/A	30	6	20	N/A	N/A	N/A	N/A
						19–20	N/A	14	5	35.70	N/A	N/A	N/A	N/A
						21+	N/A	13	1	7.70	N/A	N/A	N/A	N/A
						<18	Girls <18 years mean = 18.5 months ≥18 years mean = 9.6 months; *p* = 0.007	N/A	N/A	N/A	0.88	0.77, 1.10	0.99	(0.85, 1.15)
8	Biswas et al., 2020	India	Cross‐sectional	Women who engaged in consensual sex in exchange for cash or in‐kind payment at least once within the past 1 month	2016–2017	<20	N/A	386	18	4.66	0.64	0.37–1.09	0.79	0.38–1.62
						20+	N/A	941	67	7.11	1.0 [reference]		N/A	N/A
9	Silverman et al., 2007	Kathmandu, Nepal	Cross‐sectional analysis (medical records review)	Documented by police authorities in Indian destination cities and by short‐term care NGO as having been trafficked for sexual exploitation in brothels	January 1997–December 2005	7–14	N/A	33	20	60.6	N/A	1.51, 7.75	3.7	1.32, 10.34
						15–17	N/A	76	30	39.5	1.45	0.78, 271	1.19	0.57, 2.47
						18+ (18–32)	N/A	100	31	31.0	1.0 [ref]	N/A	1.0 [ref]	N/A
10	Wirth et al., 2013	Karnataka, India	Cross‐sectional	Having exchanged sex for money at least once in the previous month	August 2005–August 2006	<20	Mean 10.3 years (SD 12.7 years)	N/A	N/A	22.8	N/A	N/A	N/A	N/A
						20–27	Mean 4.9 years (SD 5.4 years)	N/A	N/A	15.1	N/A	N/A	N/A	N/A
						28+	Mean 4.3 years (SD 6.2 years)	N/A	N/A	12.0	N/A	N/A	N/A	N/A
11	Falb et al., 2011	Calcutta, India	Cross‐sectional analysis (medical records review)	This paper reports on victims of human sex trafficking defined in the paper as "forced, coerced, fraudulent or deceitful entry into sex work, entry by abduction or entry into such work under the age of 18"	January 1997 and December 2005	6–14	N/A	6	2	33.3	N/A	N/A	N/A	N/A
						15–17	N/A	24	4	16.7	N/A	N/A	N/A	N/A
						<18	N/A	30	6	20.0	N/A	N/A	N/A	N/A
						18+	N/A	6	2	33.3	N/A	N/A	N/A	N/A
12	van Griensven et al., 1995	Chiang Mai City and Sungai Kolok, Thailand	Cross‐sectional	Not defined but recruited directly from the venue where they perform CSW, and weekly STD check‐up required for all sex workers in Thailand	May 1992	12–15	N/A	84	30	35.7	4.43	2.37, 8.29	3.68	N/A
						16–17	N/A	167	50	29.9	3.4	2.00, 5.81	2.32	N/A
						18–20	N/A	275	67	24.4	2.57	1.57, 4.22	2	N/A
						21+	N/A	269	30	11.2	1 [ref]	N/A	1 [ref]	N/A
13	Parcesepe et al., 2016	Mombasa, Kenya	RCT (extracted baseline data)	Self‐report of exchange of any type of sex, including oral, anal or vaginal sex for money or gifts	March–September 2011	<18	8 years	162	29	17.9	N/A	N/A	N/A	N/A
						18+	4 years	654	135	20.6	N/A	N/A	N/A	N/A
14	Grosso et al., 2019	Lesotho	Cross‐sectional	Selling sex for money occasionally or full‐time within the province in the last 6 months	2014	<18	N/A	53	38	71.70	1.0 [ref]	N/A	N/A	N/A
						> = 18	N/A	262	182	69.5	1.11	0.58–2.14	N/A	N/A
15	Hernandez et al., 2019	Ecuador	Cross‐sectional	Self‐reported sex work	2010	<18	N/A	5	15.1	N/A	N/A	N/A	N/A	N/A
						> = 18	N/A	6	2.5	N/A	N/A	N/A	AOR 0.13	95% CI 0.03–0.57
16	Boyce et al., 2020	Guatemala	Cross‐sectional	Report exchanging sex for money in the last 12 months	2012–2013	<16	N/A	4.6	N/A	5.2	1.6–16.5	N/A	1.6–13.2	N/A
						16–17	N/A	4.8	N/A	5.5	1.9–16.2	N/A	2.0–17.7	N/A
						18+	N/A	0.9	N/A	Ref	1	<0.01	1	*p*<0.001
17	Tokar et al., 2019	Ukraine	Cross‐sectional	Exchanged sex for money/drugs/services or goods in the last 6 months	2013–2014	12–13	N/A	9	1	0.5	N/A	N/A	N/A	N/A
						14–18	N/A	1242	33	17.4	2.8	(0.2–28.6)	5.3	(0.2–135.0)
						19–23	N/A	2227	93	49.2	3.6	(0.3–36.5)	6.5	(0.2–155.3)
						24–28	N/A	1067	40	21.1	9.5	(0.8–101.0)	12.6	(0.5–320.6)
						29–33	N/A	252	18	9.5	28.2	(1.9–417.3)	54.8	(1.7–1709.7)
						34+	N/A	70	4	2.1	1.0 (ref)		1	ref
18	Khezri et al., 2020	Iran	Cross‐sectional	Having sold sex in exchange for livelihood (i.e. money, goods, services or drugs) with more than one client within the previous 12 months at interview time	2015	<18	N/A	131	4	7.58	1.65	(0.56, 4.88)	1.4	(0.43, 4.53)
						18+	N/A	1165	22	2.84	ref		ref	
							N/A	N/A	N/A	N/A	N/A	N/A	N/A	N/A

Overall HIV prevalence ranged from 5.2% in Iran [44] to 42.9% in Rwanda [48]. Studies were published between 1995 and 2020 with 13 studies published since 2010. The prevalence of HIV was generally higher in women who initiated selling sex at an earlier age compared to those who initiated later (*N* = 12), although four studies found comparable percentages in older initiators in Mexico, India, Ukraine and Kenya. Only four studies reported data on the duration of selling sex by age of initiation, an important source of potential confounding. Five of eight studies using measures of transactional sex found a higher HIV prevalence with initiation at younger ages. A total of 6/9 studies with measures of self‐reported sex work and 1/1 of studies with measures of survival sex reported a higher prevalence in those initiating at an earlier compared to a later age.

In a meta‐analysis of pooled prevalence estimates by age of initiation of selling sex, the prevalence of HIV among those who initiated at under 18 years of age was 28.8 (95% CI: 18.9, 38.7; Table 3 and Appendix Figure S6). There was extreme heterogeneity between studies (*X* ^2^ = 3856.68, degrees of freedom = 11, *p*<0.001, *I* ^2^ = 96.9%). Among women who initiated at 18 years or above, the pooled prevalence of HIV was 20.5 (95% CI: 12.4, 28.6; Appendix Figure S7), again with substantial heterogeneity (*X* ^2^ = 802.17, degrees of freedom = 10, *p*<0.001; *I* ^2^ = 98.8%).

**Table 3:** HIV incidence rate or prevalence with corresponding 95% confidence intervals for two meta‐analyses of HIV incidence and prevalence by age

Incidence meta‐analysis	Incidence rate (95% CI)	Heterogeneity	*I* ^2^
		*X* ^2^, df, *p*‐value	
Age			
<25	5.3 (3.5, 7.1)	38.62, 8, <0.00	79.3%
≥25	2.8 (1.7, 3.9)	47.02, 7, <0.00	85.1%
20–29	4.4 (1.3, 7.4)	222.91, 4, <0.00	98.2%
Sensitivity analysis			
≤25	5.4 (3.3, 7.4)	123.59, 10, <0.00	91.9%
>25	2.8 (1.7, 3.9)	47.02, 7, <0.00	85.1%
Prevalence meta‐analysis	Prevalence (95% CI)	Heterogeneity	*I* ^2^
		*X* ^2^, df, *p*‐value	
Age			
<18	28.8 (18.9, 38.6)	357.14, 11, <0.00	96.9%
≥18	20.5 (12.4, 28.6)	802.17, 10, <0.00	98.8%

### 3.3 Quality assessment results and risk of bias

All 12 studies identified for inclusion in the HIV incidence review were scored by two independent reviewers using the GHQAT. The overall combined median score was 12 [IQR 11, 13) out of a possible 16 points, and all studies were assessed as being of similarly “fair quality.” For the HIV prevalence review, the overall combined median score was 10 [IQR 9, 10] out of 13 total points. With the exception of one study that was rated as “poor,” all studies were judged to be of “fair” quality [40]. Results from the quality assessment are included in Appendix S4. Given the limited number of studies originally identified for inclusion, and the consistency in quality assessment scores using the GHQAT, no further changes were made to the meta‐analyses based on these findings.

## 4 DISCUSSION

This study is among the first to synthesize available literature on HIV incidence and prevalence in women who sell sex by age. These companion reviews identified 12 studies with estimates of HIV incidence stratified by age, and 18 studies with estimates of HIV prevalence stratified by age of initiation of selling sex. Most studies demonstrated a higher incidence of HIV among YWSS compared to older WSS, and a higher prevalence of HIV among those who began selling sex as a minor. In a meta‐analysis of pooled estimates by age group, HIV incidence was elevated in younger compared to older women, although CIs overlapped. Our meta‐analysis of HIV prevalence by age at initiation yielded a similar result, whereby the prevalence of HIV among those who initiated under 18 years of age was higher than those who initiated later, although CIs overlapped. Together, findings suggest that young women who sell sex may be at a higher risk of HIV than older women who sell sex and are seemingly more likely to also have a higher burden of HIV later in life.

Targeted key population programmes have historically focused on providing services for adults ages 18 years and older without a specific focus on younger adults or inclusion of those under 18, although in some settings have expanded to offer tailored HIV programming for adolescent girls and young women. YWSS face intersectional challenges and needs related to both age and being a woman who sells sex, including not only HIV prevention and treatment, but also gender‐based violence, economic challenges, mental health, housing, education and parenting needs [5, 29, 54, 55, 56]. Needs vary tremendously during the transitional period of adolescence and young adulthood, and services designed for adults will not meet all of these varied and dynamic needs. Yet, the evidence is lacking on HIV risk specifically among YWSS, and as a result, there remains a lack of clarity on how best to meet the myriad needs of YWSS, and few HIV programmes have adequately addressed this [19, 57, 58]. The elevated HIV incidence seen in this review/meta‐analysis, coupled with a high prevalence among those who began selling sex at an earlier age in this review/meta‐analysis, reinforces that YWSS are exposed to HIV early on and there is thus a brief window of time in which to reach them with prevention services [19]. The high incidence of HIV among YWSS in this review/meta‐analysis along with lower levels of access to care and viral suppression in other studies also suggests opportunities for onward transmission to clients and other partners, as the risk of onward HIV transmission is greatest for newly acquired infections [5, 29, 59, 60]. Younger female sex workers are less likely to know they were HIV positive, to report any Antiretroviral therapy (ART) exposure and to be virally suppressed compared to older female sex workers [54, 61]. Thus, minimizing the duration of time of undiagnosed and untreated HIV infection among YWSS is critical to meeting the global HIV agenda of HIV elimination.

In general, a lack of high‐quality epidemiologic data for key populations challenges programmes from meeting the essential needs of sex workers, including YWSS [62, 63]. Further, HIV programmes often do not disaggregate routinely collected data by age making it difficult to determine how many YWSS are accessing services [13]. Increased efforts are needed to routinely disaggregate and report data by meaningful age bands to ensure that programmes are sufficiently reached by YWSS. Overall, the quality of evidence in our review was moderate (graded as “good” or “fair” using the GHQAT) given that there were very few studies, most had smaller sample sizes within each age category, and there was substantial heterogeneity across studies.

We documented substantial heterogeneity across studies, although the small number of studies and inconsistencies in the reporting of key variables precluded a more nuanced analysis of these contextual differences. Heterogeneity may be due to inherent differences between study settings, measurement of selling sex, measurement of HIV outcomes or other differences. We were unable to do any sensitivity analyses by country given the heterogeneity across studies and the small number of studies. The high HIV prevalence observed in most studies may be due to higher incidence among YWSS or a longer duration of time selling sex among those who initiated earlier. With few studies reporting duration of time selling sex, we were unable to differentiate between the two. Further, those studies that focused on formal sex work generally reported a higher prevalence of HIV overall compared to those focused on transactional sex alone. This is consistent with prior literature indicating that sexual exchange occurs along a spectrum and the motivations underlying formal sex work versus transactional sex may vary leading to differences in HIV risk [11, 15, 31]. There is also evidence that those who engage in transactional sex but do not self‐identify as sex workers may be less likely to access HIV and other health services [64], and they may, therefore, be a population in particular need of targeted services.

There were other limitations to this systematic review and meta‐analysis. First, the review identified a relatively small number of studies with data on HIV incidence disaggregated by age, and on HIV prevalence by age of initiation of selling sex. In the resulting meta‐analysis, YWSS had a high pooled incidence of HIV, but CIs overlapped with those reported for older WSS. Further, due to the small sample size of included studies in the meta‐analysis, we had limited precision and limited power to fully identify differences between these groups. Second, only two regions of the world were represented in the incidence review, further limiting generalizability or transportability beyond those regions. This speaks to the need for more routine collection of data to ensure we are fully able to meet the needs of this vulnerable population on a global scale. Third, the meta‐analysis stratified estimates based on age cut‐offs that allowed for the largest number of studies to be included as well as the standard definition used to define adolescent girls as young women. However, studies often used inconsistent age bands making it difficult to pool and compare estimates. Given the range of geographic settings, age categories and definitions of selling sex, we also did not assess meta‐biases or confidence in cumulative evidence. Fourth, HIV prevalence and incidence will be influenced by HIV prevention interventions, social determinants of health and factors related to access to care that will vary by region and the year in which the study was undertaken. More data are needed on social determinants of health and access to care across studies to better understand these contextual factors. Fifth, we did not do a comprehensive review of the grey literature and may have missed information that was not published in peer‐reviewed journals.

## 5 CONCLUSIONS

These companion reviews offer an important perspective on the relative HIV risk of engaging in selling sex at a young age, as compared to older WSS. We identified an increased burden of HIV among YWSS, with generally higher HIV incidence compared to older WSS, and higher HIV prevalence in women who initiated selling sex at an earlier age, although CIs did overlap. Our findings highlight the unique challenges YWSS face, and the importance of ensuring that service delivery addresses their specific needs. Research and programming should routinely stratify data into meaningful age bands to differentiate and intervene within this population. Ensuring we meet their needs is a public health imperative and critical to meeting our global HIV goals.

## COMPETING INTERESTS

The authors declare no competing interests.

## AUTHORS’ CONTRIBUTIONS

MCDS, KBR and SN conceptualized and designed the study. MCDS and KBR searched and reviewed all studies for both literature reviews. CL did the meta‐analysis for incidence and prevalence. MCDS led writing but all authors contributed to the writing and reviewed and edited the manuscript.

## FUNDING

This study was funded by the NIH through K01MH110316, K01MH129226 and R21HD106583. CL was supported by the National Institute of Mental Health under Award Number F31MH128079.

## Supporting information


**Appendix S1**: Appendix figures.

**Appendix S2**: PRISMA 2009 checklist.

**Appendix S3**: The final search strategies.

**Appendix S4**: Results from the quality assessment.

## Data Availability

Data are contained within the manuscript or available by contacting the corresponding author.
