# Supplementary material for: Differentiating the incidence and burden of HIV by age among women who sell sex: a systematic review and meta‐analysis
Source: J Int AIDS Soc. 2022 Oct 27;25(10):e26028. doi: 10.1002/jia2.26028 (PMC9612831; doi:10.1002/jia2.26028)
Supplement: Supplementary file 1 — Appendix S1: Appendix figures. [file JIA2-25-e26028-s003.docx]

**Appendix Figure 1: Meta-analysis of incidence rate for YWSS less than 25 years old**

**Appendix Figure 2: Sensitivity analysis, meta-analysis of incidence rate for YWSS ≤25 years old with estimates from two additional studies added**

**Appendix Figure 3: Meta-analysis of incidence rate for YWSS ≥25 years old**

**Appendix Figure 4: Sensitivity analysis, meta-analysis of incidence rate for YWSS >25 years with estimates from two additional studies added**

**Appendix Figure 5: Meta-analysis of incidence rate for YWSS 20-29 years old**

**Appendix Figure 6: Meta-analysis of prevalence for YWSS who initiate sex work <18 years old**

**Appendix Figure 7: Meta-analysis of prevalence for YWSS who initiate sex work ≥ 18 years**
