# Supplementary material for: Differentiating the incidence and burden of HIV by age among women who sell sex: a systematic review and meta‐analysis
Source: J Int AIDS Soc. 2022 Oct 27;25(10):e26028. doi: 10.1002/jia2.26028 (PMC9612831; doi:10.1002/jia2.26028)
Supplement: Supplementary file 3 — Appendix S3: The final search strategies. [file JIA2-25-e26028-s002.docx]

**Searches Update_strategy_1-30-2020**

**Review #1 Young FSW & Incident HIV**

**PubMed**

(youth OR young* OR adolescent* OR teen* OR underage OR juvenile* OR child* OR minor*) AND (female* OR women OR woman OR girl*) AND (sex work* OR prostitute* OR prostitution OR sex traffick* OR "commercial sex" OR "transactional sex" OR "sex industry" OR "sex tourism" OR "sex trade" OR sell* sex OR "paid sex" OR "sex for money" OR "survival sex" OR "sexual exploitation" OR "exchange sex") AND (incidence OR incident) AND (HIV OR AIDS OR "human immunodeficiency virus" OR "acquired immunodeficiency syndrome" OR HIV/AIDS) Filters: Publication date from 2017/06/08; English 409

**Embase**

(youth OR young* OR adolescent* OR teen* OR underage OR juvenile* OR child* OR minor*) AND (female* OR wom?n OR girl*) AND ("sex work*" OR prostitute* OR prostitution OR "sex traffick*" OR "commercial sex" OR "transactional sex" OR "sex industry" OR "sex tourism" OR "sex trade" OR "sell* sex" OR "paid sex" OR "sex for money" OR "survival sex" OR "sexual exploitation" OR "exchange sex") AND (incidence OR incident) AND (HIV OR AIDS OR "human immunodeficiency virus" OR "acquired immunodeficiency syndrome" OR "HIV AIDS") AND [english]/lim AND [8-6-2017]/sd AND [2017-2020]/py 94

**Web of Science**

(youth OR young* OR adolescent* OR teen* OR underage OR juvenile* OR child* OR minor*) AND (female* OR women OR woman OR girl*) AND ("sex work*" OR prostitute* OR prostitution OR "sex traffick*" OR "commercial sex" OR "transactional sex" OR "sex industry" OR "sex tourism" OR "sex trade" OR "sell* sex" OR "paid sex" OR "sex for money" OR "survival sex" OR "sexual exploitation" OR "exchange sex") AND (incidence OR incident) AND (HIV OR AIDS OR "human immunodeficiency virus" OR "acquired immunodeficiency syndrome" OR "HIV/AIDS") AND LANGUAGE: (English) Indexes=SCI-EXPANDED, SSCI, CPCI-S, CPCI-SSH Timespan=2017-2020 (June 8, 2017-present) 34

**Sociological Abstracts**

(youth OR young* OR adolescent* OR teen* OR underage OR juvenile* OR child* OR minor*) AND (female* OR women OR woman OR girl*) AND ((sex work*) OR prostitute* OR prostitution OR (sex traffick*) OR "commercial sex" OR "transactional sex" OR "sex industry" OR "sex tourism" OR "sex trade" OR (sell* sex) OR "paid sex" OR "sex for money" OR "survival sex" OR "sexual exploitation" OR "exchange sex") AND (incidence OR incident) AND (HIV OR AIDS OR "human immunodeficiency virus" OR "acquired immunodeficiency syndrome" OR HIV/AIDS) English; June 8, 2017 – 2020 2

**PubMed**

((youth OR young* OR adolescent* OR teen* OR underage OR juvenile* OR child* OR minor*) AND (female* OR women OR woman OR girl*) AND (sex work* OR prostitute* OR prostitution OR sex traffick* OR "commercial sex" OR "transactional sex" OR "sex industry" OR "sex tourism" OR "sex trade" OR sell* sex OR "paid sex" OR "sex for money" OR "survival sex" OR "sexual exploitation" OR "exchange sex") AND (incidence OR incident) AND (HIV OR AIDS OR "human immunodeficiency virus" OR "acquired immunodeficiency syndrome" OR HIV/AIDS) AND ("1980/01/01"[Date - Publication] : "2021/02/12"[Date - Publication])) NOT "english"[Language] 194

**Embase**

((youth OR young* OR adolescent* OR teen* OR underage OR juvenile* OR child* OR minor*) AND (female* OR wom?n OR girl*) AND ("sex work*" OR prostitute* OR prostitution OR "sex traffick*" OR "commercial sex" OR "transactional sex" OR "sex industry" OR "sex tourism" OR "sex trade" OR "sell* sex" OR "paid sex" OR "sex for money" OR "survival sex" OR "sexual exploitation" OR "exchange sex") AND (incidence OR incident) AND (HIV OR AIDS OR "human immunodeficiency virus" OR "acquired immunodeficiency syndrome" OR "HIV AIDS") AND [embase]/lim AND [1-1-1980]/sd NOT [13-2-2021]/sd AND [1980-2021]/py) NOT [english]/lim 6

**Web of Science**

TS=((youth OR young* OR adolescent* OR teen* OR underage OR juvenile* OR child* OR minor*) AND (female* OR women OR woman OR girl*) AND ("sex work*" OR prostitute* OR prostitution OR "sex traffick*" OR "commercial sex" OR "transactional sex" OR "sex industry" OR "sex tourism" OR "sex trade" OR "sell* sex" OR "paid sex" OR "sex for money" OR "survival sex" OR "sexual exploitation" OR "exchange sex") AND (incidence OR incident) AND (HIV OR AIDS OR "human immunodeficiency virus" OR "acquired immunodeficiency syndrome" OR "HIV/AIDS")) NOT LA=(English) Timespan: 1980-01-01 to 2021-02-12 (Publication Date) 1

**Sociological Abstracts**

#1 (youth OR young* OR adolescent* OR teen* OR underage OR juvenile* OR child* OR minor*) AND (female* OR women OR woman OR girl*) AND ((sex work*) OR prostitute* OR prostitution OR (sex traffick*) OR "commercial sex" OR "transactional sex" OR "sex industry" OR "sex tourism" OR "sex trade" OR (sell* sex) OR "paid sex" OR "sex for money" OR "survival sex" OR "sexual exploitation" OR "exchange sex") AND (incidence OR incident) AND (HIV OR AIDS OR "human immunodeficiency virus" OR "acquired immunodeficiency syndrome" OR HIV/AIDS) Additional limits - Date: From January 01 1980 to February 12 2021; Language: English 18

#2 (youth OR young* OR adolescent* OR teen* OR underage OR juvenile* OR child* OR minor*) AND (female* OR women OR woman OR girl*) AND ((sex work*) OR prostitute* OR prostitution OR (sex traffick*) OR "commercial sex" OR "transactional sex" OR "sex industry" OR "sex tourism" OR "sex trade" OR (sell* sex) OR "paid sex" OR "sex for money" OR "survival sex" OR "sexual exploitation" OR "exchange sex") AND (incidence OR incident) AND (HIV OR AIDS OR "human immunodeficiency virus" OR "acquired immunodeficiency syndrome" OR HIV/AIDS) Additional limits - Date: From January 01 1980 to February 12 2021 18

#3 #2 NOT #1 0

**Review #2 Early Initiation FSW & HIV**

**PubMed**

(onset OR initiation OR initiator* OR entry OR (start* age) OR (sex traffick* age)) AND (female* OR women OR woman OR girl*) AND (sex work* OR prostitute* OR prostitution OR sex traffick* OR "commercial sex" OR "transactional sex" OR "sex industry" OR "sex tourism" OR "sex trade" OR sell* sex OR "paid sex" OR "sex for money" OR "survival sex" OR "sexual exploitation" OR "exchange sex") AND (HIV OR AIDS OR "human immunodeficiency virus" OR "acquired immunodeficiency syndrome" OR HIV/AIDS) Filters: Publication date from 2017/06/08; English 68

**Embase**

(onset OR initiation OR initiator* OR entry OR (start* age) OR (sex traffick* age)) AND (female* OR wom?n OR girl*) AND ("sex work*" OR prostitute* OR prostitution OR "sex traffick*" OR "commercial sex" OR "transactional sex" OR "sex industry" OR "sex tourism" OR "sex trade" OR "sell* sex" OR "paid sex" OR "sex for money" OR "survival sex" OR "sexual exploitation" OR "exchange sex") AND (HIV OR AIDS OR "human immunodeficiency virus" OR "acquired immunodeficiency syndrome" OR "HIV AIDS") AND [english]/lim AND [8-6-2017]/sd AND [2017-2020]/py 110

**Web of Science**

(onset OR initiation OR initiator* OR entry OR (start* age) OR (sex traffick* age)) AND (female* OR women OR woman OR girl*) AND ("sex work*" OR prostitute* OR prostitution OR "sex traffick*" OR "commercial sex" OR "transactional sex" OR "sex industry" OR "sex tourism" OR "sex trade" OR "sell* sex" OR "paid sex" OR "sex for money" OR "survival sex" OR "sexual exploitation" OR "exchange sex") AND (HIV OR AIDS OR "human immunodeficiency virus" OR "acquired immunodeficiency syndrome" OR "HIV/AIDS") AND LANGUAGE: (English) Indexes=SCI-EXPANDED, SSCI, CPCI-S, CPCI-SSH Timespan=2017-2020 (June 8, 2017-present) 65

**Sociological Abstracts**

(onset OR initiation OR initiator* OR entry OR (start* age) OR (sex traffick* age)) AND (female* OR women OR woman OR girl*) AND ((sex work*) OR prostitute* OR prostitution OR (sex traffick*) OR "commercial sex" OR "transactional sex" OR "sex industry" OR "sex tourism" OR "sex trade" OR (sell* sex) OR "paid sex" OR "sex for money" OR "survival sex" OR "sexual exploitation" OR "exchange sex") AND (HIV OR AIDS OR "human immunodeficiency virus" OR "acquired immunodeficiency syndrome" OR HIV/AIDS) English; June 8, 2017 – 2020 2

**PubMed**

((onset OR initiation OR initiator* OR entry OR (start* age) OR (sex traffick* age)) AND (female* OR women OR woman OR girl*) AND (sex work* OR prostitute* OR prostitution OR sex traffick* OR "commercial sex" OR "transactional sex" OR "sex industry" OR "sex tourism" OR "sex trade" OR sell* sex OR "paid sex" OR "sex for money" OR "survival sex" OR "sexual exploitation" OR "exchange sex") AND (HIV OR AIDS OR "human immunodeficiency virus" OR "acquired immunodeficiency syndrome" OR HIV/AIDS) AND ("1980/01/01"[Date - Publication] : "2021/02/12"[Date - Publication])) NOT "english"[Language] 50

**Embase**

((onset OR initiation OR initiator* OR entry OR (start* age) OR (sex traffick* age)) AND (female* OR wom?n OR girl*) AND ("sex work*" OR prostitute* OR prostitution OR "sex traffick*" OR "commercial sex" OR "transactional sex" OR "sex industry" OR "sex tourism" OR "sex trade" OR "sell* sex" OR "paid sex" OR "sex for money" OR "survival sex" OR "sexual exploitation" OR "exchange sex") AND (HIV OR AIDS OR "human immunodeficiency virus" OR "acquired immunodeficiency syndrome" OR "HIV AIDS") AND [embase]/lim AND [1-1-1980]/sd NOT [13-2-2021]/sd AND [1980-2021]/py) NOT [english]/lim 4

**Web of Science**

TS=((onset OR initiation OR initiator* OR entry OR (start* age) OR (sex traffick* age)) AND (female* OR women OR woman OR girl*) AND ("sex work*" OR prostitute* OR prostitution OR "sex traffick*" OR "commercial sex" OR "transactional sex" OR "sex industry" OR "sex tourism" OR "sex trade" OR "sell* sex" OR "paid sex" OR "sex for money" OR "survival sex" OR "sexual exploitation" OR "exchange sex") AND (HIV OR AIDS OR "human immunodeficiency virus" OR "acquired immunodeficiency syndrome" OR "HIV/AIDS")) NOT LA=(English) Timespan: 1980-01-01 to 2021-02-12 (Publication Date) 3

**Sociological Abstracts**

#1 (onset OR initiation OR initiator* OR entry OR (start* age) OR (sex traffick* age)) AND (female* OR women OR woman OR girl*) AND ((sex work*) OR prostitute* OR prostitution OR (sex traffick*) OR "commercial sex" OR "transactional sex" OR "sex industry" OR "sex tourism" OR "sex trade" OR (sell* sex) OR "paid sex" OR "sex for money" OR "survival sex" OR "sexual exploitation" OR "exchange sex") AND (HIV OR AIDS OR "human immunodeficiency virus" OR "acquired immunodeficiency syndrome" OR HIV/AIDS) Additional limits - Date: From January 01 1980 to February 12 2021; Language: English 55

#2 (onset OR initiation OR initiator* OR entry OR (start* age) OR (sex traffick* age)) AND (female* OR women OR woman OR girl*) AND ((sex work*) OR prostitute* OR prostitution OR (sex traffick*) OR "commercial sex" OR "transactional sex" OR "sex industry" OR "sex tourism" OR "sex trade" OR (sell* sex) OR "paid sex" OR "sex for money" OR "survival sex" OR "sexual exploitation" OR "exchange sex") AND (HIV OR AIDS OR "human immunodeficiency virus" OR "acquired immunodeficiency syndrome" OR HIV/AIDS) Additional limits - Date: From January 01 1980 to February 12 2021 55

#3 #2 NOT 1 0
