# Supplementary material for: Differentiating the incidence and burden of HIV by age among women who sell sex: a systematic review and meta‐analysis
Source: J Int AIDS Soc. 2022 Oct 27;25(10):e26028. doi: 10.1002/jia2.26028 (PMC9612831; doi:10.1002/jia2.26028)
Supplement: Supplementary file 4 — Appendix S4: Results from the quality assessment. [file JIA2-25-e26028-s004.docx]

**Appendix 4,**

**Appendix Table 1:** Quality Assessment Results for HIV incidence Review

| **REFERENCE** | **QA (GOOD, FAIR, POOR)** | **SCORE TOTAL** | | DESIGN | | IMPLEMENTATION | | SCORE INCIDENCE | |
| --- | --- | --- | --- | --- | --- | --- | --- | --- | --- |
|  |  | **Reviewer 1** | **Reviewer 2** | Reviewer 1 | Reviewer 2 | Reviewer 1 | Reviewer 2 | Reviewer 1 | Reviewer 2 |
| Vandepitte, J. et al | Fair | 12 | 15 | 2 | 4 | 2 | 3 | 8 | 8 |
| Braunstein, SL. et al | Fair | 12 | 13 | 3 | 3 | 2 | 1 | 7 | 9 |
| Gray, JA. et al | Fair | 11 | 12 | 3 | 3 | 0 | 3 | 8 | 6 |
| Hargreaves, JR. et al | Fair | 13 | 8 | 3 | 3 | 2 | 0 | 8 | 5 |
| Kanki, PJ. et al | Fair | 12 | 13 | 3 | 3 | 0 | 1 | 9 | 9 |
| Kilburn, K. et al | Fair | 11 | 12 | 3 | 3 | 0 | 0 | 8 | 9 |
| Naicker, N. et al | Fair | 12 | 13 | 3 | 3 | 1 | 2 | 8 | 8 |
| Kilmarx, PH. et al | Fair | 12 | 11 | 2 | 2 | 2 | 0 | 8 | 9 |
| Morineau, G. et al | Fair | 15 | 12 | 4 | 3 | 3 | 1 | 8 | 8 |
| Saphonn, V. et al | Fair | 12 | 13 | 3 | 3 | 2 | 2 | 7 | 8 |
| Couture, MC. et al | Fair | 10 | 11 | 2 | 3 | 2 | 1 | 6 | 7 |
| Su. et al | Fair | 11 | 11 | 3 | 3 | 2 | 0 | 6 | 8 |

**Appendix , table 2:** Quality Assessment Results for HIV prevalence Review

| **REFERENCE** | **QA (GOOD, FAIR, POOR)** | **SCORE TOTAL** | | DESIGN | | IMPLEMENTATION | | SCORE PREVALENCE | |
| --- | --- | --- | --- | --- | --- | --- | --- | --- | --- |
|  |  | **Reviewer 1** | **Reviewer 2** | Reviewer 1 | Reviewer 2 | Reviewer 1 | Reviewer 2 | Reviewer 1 | Reviewer 2 |
| Shannon, K. et al | Fair | 9 | 8 | 3 | 3 | 2 | 0 | 4 | 5 |
| Goldenberg, SM. et al | Fair | 11 | 8 | 3 | 3 | 3 | 1 | 5 | 4 |
| Hernandez, I. et al | Fair | 10 | 10 | 4 | 4 | 2 | 1 | 4 | 5 |
| Tokar, A. et al | Fair | 13 | 11 | 4 | 4 | 3 | 1 | 6 | 6 |
| Falb, KL. et al | Poor | 9 | 2 | 2 | 1 | 3 | 0 | 4 | 1 |
| Footer, KHA. et al | Fair | 10 | 8 | 3 | 3 | 3 | 0 | 4 | 5 |
| Boyce, SC. et al | Fair | 10 | 10 | 2 | 3 | 2 | 1 | 6 | 6 |
| Biswas, S. et al | Fair | 12 | 8 | 3 | 3 | 3 | 0 | 6 | 5 |
| Khezri, M. et al | Fair | 10 | 12 | 3 | 3 | 1 | 3 | 6 | 6 |
| Van Griensven, GJP. et al | Fair | 11 | 9 | 4 | 3 | 1 | 0 | 6 | 6 |
| Goldenberg, SM. et al | Fair | 10 | 9 | 3 | 3 | 1 | 0 | 6 | 6 |
| Surratt, HL. et al | Fair | 9 | 6 | 3 | 3 | 1 | 0 | 5 | 3 |
| Limpakarnjanarat, K. et al | Fair | 9 | 11 | 3 | 3 | 1 | 3 | 5 | 5 |
| Silverman, JG. et al | Fair | 7 | 8 | 3 | 2 | 0 | 1 | 4 | 5 |
| Silverman, JG. et al | Fair | 7 | 8 | 3 | 2 | 1 | 1 | 3 | 5 |
| Wirth, KE. et al | Fair | 12 | 11 | 3 | 3 | 3 | 2 | 6 | 6 |
| Parcesepe, AM. et al | Fair | 9 | 10 | 3 | 2 | 1 | 3 | 5 | 5 |
| Grosso, A. et al | Fair | 11 | 11 | 4 | 4 | 1 | 3 | 6 | 4 |
